# Self-rated health and sociodemographic inequalities among Venezuelan adults: a study based on the *National Survey of Living Conditions* (ENCOVI 2021)

**DOI:** 10.1590/0102-311XEN149323

**Published:** 2024-06-21

**Authors:** Dalia Elena Romero, Anitza Freitez, Leo Ramos Maia, Nathalia Andrade de Souza

**Affiliations:** 1 Instituto de Comunicação e Informação Científica e Tecnológica em Saúde, Fundação Oswaldo Cruz, Rio de Janeiro, Brasil.; 2 Instituto de Investigaciones Económicas y Sociales, Universidad Católica Andrés Bello, Caracas, Venezuela.; 3 Escola Nacional de Saúde Pública Sérgio Arouca, Fundação Oswaldo Cruz, Rio de Janeiro, Brasil.

**Keywords:** Self-Testing, Sanitary Condition, Health Surveys, Autoevaluación, Condición Sanitaria, Encuestas de Salud

## Abstract

Self-rated health is an indicator that can be easily identified in health surveys, widely used to measure physical, social, mental, and health aspects of the population, and predict premature mortality. In Venezuela, this information only began to be collected recently, in the *National Survey of Living Conditions* (ENCOVI). In this context, our study aims to analyze the demographic and socioeconomic factors associated with non-positive self-rated health among Venezuelan adults. The ENCOVI 2021 (n = 16,803) was used as a data source, assessing a probability stratified sample with questions about health, education, emigration, and other social and economic aspects. Crude and adjusted prevalence ratio analyses were performed using Poisson regression models with robust variance. The prevalence of fair/bad self-rated health among Venezuelans was 17.8%. The results indicated a strong association between outcome prevalence and age group, 3.81 times higher (95%CI: 3.29-4.41) among individuals aged 60 or more when compared to individuals aged 18 to 29 years. Also, participants experiencing severe food insecurity had a prevalence 2 times higher (95%CI: 1.61-2.47) than those who did not have any level of food insecurity. Factors such as poverty, education, recent emigration of family members, and sex also showed a significant influence, also when analyzed independently. The results show that special attention should be dedicated to the health of individuals facing hunger and of the older people.

## Resumen

La autoevaluación de salud es un indicador de simple captación en encuestas de salud, muy utilizado en investigaciones para medir aspectos físicos, sociales, mentales y de salud de la población, además de predecir la mortalidad precoz. En el caso de los venezolanos, esta información recién comenzó a recopilarse en la *Encuesta Nacional de Condiciones de Vida* (ENCOVI). En este contexto, el objetivo del estudio es analizar los factores demográficos y socioeconómicos asociados con la autoevaluación de salud no positiva entre los venezolanos adultos. Se utiliza la ENCOVI 2021 como fuente de datos (n = 16.803), que tiene una muestra probabilística y estratificada, además de preguntas sobre salud, educación, migración y otros aspectos sociales y económicos. Se realizaron análisis de la razón de prevalencia crudos y ajustados, estimados a través de modelos de regresión de Poisson con varianza robusta. La prevalencia de la autoevaluación de salud regular/mala entre los venezolanos fue del 17,8%. Los resultados mostraron una fuerte asociación entre la prevalencia del resultado y el grupo de edad, siendo 3,81 veces mayor (IC95%: 3,29-4,41) entre las personas con 60 años o más, en comparación con las de 18 a 29 años. Además, los participantes en situación de inseguridad alimentaria grave presentaron una prevalencia 2 veces mayor (IC95%: 1,61-2,47) que aquellos que no enfrentaron ningún nivel de inseguridad alimentaria. Factores como pobreza, escolaridad, emigración reciente de familiares y género también demostraron una influencia significativa, aun cuando analizados de manera independiente. Los resultados resaltan la necesidad de prestar especial atención a la salud de los que enfrentan hambre y de las personas mayores.

## Introduction

Self-rated health is an indicator that can be easily identified in surveys, used to express a subjective assessment of health status, also acting as a proxy for the objective characteristics of an individual’s health ^1^. It covers physical and social, mental and health dimensions. This indicator is also used as a predictor of premature mortality, which has attracted considerable interest in the scientific community since the end of the 20th century ^2^ ^,^ ^3^.

Social and economic circumstances have an impact on our physical and mental health, and this is why the relationship between self-rated health and demographic and socioeconomic factors has been documented ^4^ ^,^ ^5^ ^,^ ^6^ ^,^ ^7^. Although this relationship varies in strength between countries ^4^, in general, the association of worse self-rated health and female individuals ^5^ ^,^ ^7^, older people ^5^ ^,^ ^7^, poorer individuals ^4^ ^,^ ^6^ ^,^ ^7^, lower educational level ^4^ ^,^ ^5^ ^,^ ^7^, and in a situation of food insecurity ^6^ is well documented in the literature.

On the other hand, the influence of family member emigration on self-rated health in situations of emigration crisis has been underestimated. Most studies on self-rated health and emigration focus on assessing the condition of emigrants, neglecting the impact of emigration on family members who remain in the country of origin ^8^. Studies conducted in these contexts of crisis must consider that emigration fragments the family nucleus, generating more responsibility for family care to few people, which can affect their health.

In Venezuela, the collection of information about self-rated health began recently, in a national survey titled *National Survey of Living Conditions* (Encuesta Nacional de Condiciones de Vida - ENCOVI) ^9^ conducted during the COVID-19 pandemic. An international study that assessed self-rated health and included Venezuela was conducted between 2003 and 2007, but focused only on the elderly population (n = 2,026) ^10^. The authors showed that older people in Venezuela had a favorable self-rated health when compared to other eight countries analyzed ^7^.

Extreme poverty has increased in Venezuela, from 9.3% to 76.6% between 2013 and 2021 ^11^. Emigration has also been intense, between 2.3 million and 4 million Venezuelan migrants since 2015 ^12^. In this context of acute humanitarian crisis, which has also influenced the deterioration of the health system ^13^, evidence has been documented of outbreaks of preventable infectious diseases ^14^, lack of vaccine and medication ^13^, and hospitalizations and deaths due to malnutrition ^15^. These findings indicate the health conditions of Venezuelans have declined.

For years, Venezuela has restricted the production and dissemination of information, including epidemiological data ^12^. To promote official information sources, in 2014 Venezuelan research institutions started to conduct the ENCOVI ^9^. The study, which has a cross-sectional, multithematic design, national coverage and annual frequency, has become one of the most used sources of information for articles focused on the living and health conditions of the Venezuelan population ^16^.

Learning about the subjective perception of the population health is essential in a country with serious economic and emigration problems and the unavailability of public health information. Reliable information that is regularly released and publicly available provides answers to the population’s main problems, becoming a required condition for scientific evidence, supporting the implementation of effective health actions and monitoring of public policies ^17^. In this sense, this article assessed the demographic and socioeconomic factors associated with non-positive self-rated health among Venezuelan adults.

## Methods

### Data sources and sample

Venezuela is a country on the northern coast of South America. According to the United Nations (UN) Population Division, Venezuela had 28,436,000 inhabitants in 2021. The country has faced significant economic and political challenges in recent years, which have impacted its socioeconomic and demographic situation ^16^.

This study has a population-based cross-sectional design using data from the ENCOVI survey conducted in Venezuela between February and April 2021, and coordinated by the Institute of Economic and Social Research (IIES, acronym in Spanish) of the Andrés Bello Catholic University (UCAB, acronym in Spanish). ENCOVI 2021 has questions related to education, health, nutrition, and access to food, poverty, unemployment, and emigration of Venezuelans.

The survey uses a 3-stage probability conglomerate sample, based on per capita family income, obtained through ENCOVI 2019/2020 at state level. The questionnaire had 21 sections and 36 subsections and, in total, 788 questions. The questions were applied to each household and all household members. More information about ENCOVI 2021 can be obtained in the research technical document ^9^.

The final sample of 42,444 people was calibrated with weights that considered data from the 2011 census and population projections for 2021 of the 2019 revision of the UN World Population Prospects.

Our study considered adult individuals aged ≥ 18 years who answered the question: “In the last 12 months, how do you consider the health status of the respondent? (n = 16,803).

### Variables

The outcome of interest in this study was non-positive self-rated health. To assess it, the question “In the last 12 months, how do you consider the health status of the respondent?” was used, which had the response options: “very good”, “good”, “fair”, and “bad”. The last two options were grouped for the outcome definition. This categorization is commonly used in similar studies, which allows comparisons between them ^6^ ^,^ ^18^ ^,^ ^19^ ^,^ ^20^ ^,^ ^21^.

The demographic characteristics associated with the outcome were sex (male or female) and age groups (young adults: 18 to 29 years old, adults: 30 to 59 years old, and older people: 60 years old or more).

The socioeconomic factors considered in this study were:

Educational level, assessed with the following question: “What was the last educational level in which the interviewee completed a grade or a school year, semester or quarter?” with the response options: complete primary education or less; incomplete high school; complete high school; and incomplete or complete higher education.

Level of monetary poverty estimated in ENCOVI 2021 based on per capita household income related to the amount defined in the regulatory food basket (Canasta Alimentaria Normativa - CAN), whose amount covers the minimum calorie needs ^22^. In cases where per capita income is higher than or equal to twice the CAN amount per capita, the respondent is considered “not poor”. For per capita income higher than or equal to the CAN amount, but less than twice, the respondent is considered “not extremely poor”. For per capita income lower than the CAN amount, the respondent is considered “extremely poor”.

Food Insecurity Scale estimated in ENCOVI 2021 based on the *Latin American and Caribbean Food Security Scale* (Escala Latinoamericana y Caribeña de Seguridad Alimentaria - ELCSA) ^23^. The ELCSA has 15 questions that address situations experienced by the respondents in the 3-month period before the interview related to the quantity and quality of foods available and their strategies to handle the lack of food. The responses were: severe food insecurity; moderate food insecurity; mild food insecurity; no food insecurity. Respondents with severe food insecurity were considered in situation of hunger.

Parental emigration defined as the recent emigration of a parent, identified in the question: “In the last 5 years, since January 2016, has any person who lives or lived with you in your home moved to another country?” with yes or no as possible responses.

### Analysis

First, study population was characterized according to the variables considered in the study through percentage distribution and respective 95% confidence intervals (95%CI). Then, the prevalence of fair/bad self-rated health was estimated according to demographic and socioeconomic characteristics. The association between the variables was assessed by Pearson’s chi-square test with Rao-Scott correction, considering a significance level of 5%.

In the analysis of factors independently associated with non-positive self-rated health, the following variables were considered as they showed a significant association with the outcome in the bivariate analysis: sex, age group, education, level of monetary poverty, food insecurity, and parental emigration. Prevalence ratios (PR) and 95%CI were estimated using Poisson regression models with robust variance. The analyses were conducted using the SPSS 21 statistical package (https://www.ibm.com/), considering the sample weight obtained for sample calibration.

### Ethical aspects

As this is a study using exclusively secondary data of public domain, it is exempt from the approval by the Human Research Ethics Committee, according to *Resolution n. 466/2012* of the Brazilian National Health Council.

## Results

The study population had mostly female individuals (64.8%; 95%CI: 64.0-65.5) and people aged 30 to 59 years (60.3%; 95%CI: 59.5-61.1) (Table 1).

**Table 1:** Percentage distribution of the Venezuelan adult population according to demographic and socioeconomic characteristics. Venezuela, 2021.

Variables	n	%	95%CI
Sex			
Male	5,471	35.2	34.5-36.0
Female	10,066	64.8	64.0-65.5
Age (years)			
18-29	2,895	18.6	18.0-19.2
30-59	9,368	60.3	59.5-61.1
60 or more	3,275	21.1	20.4-21.7
Education			
No education	60	0.4	0.3-0.5
Incomplete primary education	1,505	10.1	9.6-10.6
Complete primary education	2,187	14.7	14.1-15.3
Incomplete high school	2,321	15.6	14.7-16.2
Complete high school	4,642	31.2	30.5-32
Higher education - technical course (complete or incomplete)	1,184	8.0	7.5-8.4
Higher education - university course (complete or incomplete)	2,963	19.9	19.3-20.6
Level of monetary poverty			
Not poor	1,317	8.5	8.1-9.5
Not extreme poverty	3,480	22.6	21.9-23.2
Extreme poverty	10,631	68.9	68.2-69.6
Food Insecurity Scale			
No food insecurity	1,093	7.0	6.6-7.4
Mild food insecurity	5,264	33.9	33.2-35.1
Moderate food insecurity	5,172	33.3	32.6-34.1
Severe food insecurity	3,992	25.7	25.0-26.4
Parental emigration			
No	14,105	90.8	90.4-91.3
Yes	1,425	9.2	8.7-9.6
Self-rated health			
Very good	6,011	38.7	38.0-39.4
Good	6,763	43.5	42.9-44.2
Fair	2,554	16.4	16.0-16.9
Poor	209	1.3	1.2-1.5

95%CI: 95% confidence interval.

Source: *National Survey of Living Conditions* (ENCOVI 2021) ^9^.

As seen in Table 1, a large part of the Venezuelan population presented poor socioeconomic conditions. More than 90% showed some level of monetary poverty or food insecurity. Extreme cases were also observed: almost 7 out of 10 Venezuelans were in extreme poverty (68.9%; 95%CI: 68.2-69.6) while 25.7% (95%CI: 25.0-26.4) showed severe food insecurity, i.e., they faced hunger.

Around 80% of Venezuelan adults assessed their health positively: 38.7% (95%CI: 38.0-39.4) rated it as very good and 43.5% (95%CI: 42.9-44, 2) as good (Table 1).

Table 2 shows the prevalence of fair/bad self-rated health according to demographic and socioeconomic characteristics. Of the total number of Venezuelan adults, 17.8% (95%CI: 17.2-18.4) reported fair/bad self-rated health. The groups that assessed their health negatively were: older people (32.9%; 95%CI: 31.3-34.5), people who only completed primary education (24.6%; 95%CI: 23.2-25.9), people with severe food insecurity (23.8%; 95%CI: 22.5-25.1), and people with recently emigrated family members (22.7%; 95%CI: 20.5-24.9). On the other hand, young adults and people without food insecurity had a prevalence of fair/bad self-rated health below 10% (Table 2).

**Table 2:** Prevalence of Venezuelan adults with fair/poor self-rated health, according to demographic and socioeconomic characteristics. Venezuela, 2021.

Variables	%	95%CI	p-value
Total	17.8	17.2-18.4	
Sex			< 0.001
Male	14.9	14.0-15.8	
Female	19.4	18.6-20.1	
Age (years)			< 0.001
18-29	7.7	6.7-8.7	
30-59	15.6	14.9-16.3	
60 or more	32.9	31.3-34.5	
Education			< 0.001
Complete primary education or less	24.6	23.2-25.9	
Incomplete high school	19.1	17.5-20.7	
Complete high school	15.0	13.9-16.0	
Incomplete higher education or more	12.3	11.3-13.3	
Level of monetary poverty			< 0.001
Not poor	12.6	10.8-14.4	
Not extreme poverty	17.0	15.8-18.2	
Extreme poverty	18.7	17.9-19.4	
Food Insecurity Scale			< 0.001
No food insecurity	9.4	7.7-11.2	
Mild food insecurity	12.7	11.8-13.6	
Moderate food insecurity	20.0	18.9-21.1	
Severe food insecurity	23.8	22.5-25.1	
Parental emigration			< 0.001
No	17.3	16.7-17.9	
Yes	22,7	20.5-24.9	

95%CI: 95% confidence interval.

Source: *National Survey of Living Conditions* (ENCOVI 2021) ^9^.

In the analysis of factors independently associated with non-positive self-rated health (Table 3), being a female individual, being older, having a lower educational level, having a higher level of poverty and food insecurity, and having a family member who emigrated recently were factors associated with the outcome. In adjusted analysis, two factors were more associated with fair/bad self-rated health: age and food insecurity; among the elderly, a negative perception of health was about four times higher in relation to younger people (PR = 3.81; 95%CI: 3.29-4.41) and, among individuals in situations of severe food insecurity, this perception was twice as high when compared to those who did not present any level of food insecurity (PR = 2.00; 95%CI: 1.61-2.47).

**Table 3:** Crude and adjusted prevalence ratios (PR) of fair/poor self-rated health, according to demographic and socioeconomic characteristics in adults. Venezuela, 2021.

Variables	Crude PR	95%CI	p-value	Adjusted PR	95%CI	p-value
Sex						
Male	1.41	1.31-1.53	< 0.001	1.35	1.25-1.47	< 0.001
Female	1.00	-	-	1.00		
Age (years)						
18-29	4.10	3.57-4.71	< 0.001	3.81	3.29-4.41	< 0.001
30-59	1.97	1.72-2.25	< 0.001	1.91	1.66-2.20	< 0.001
60 or more	1.00	-	-	1.00		
Education						
Complete primary education or less	1.45	1.31-1.62	< 0.001	1.36	1.22-1.53	< 0.001
Incomplete high school	1.57	1.39-1.76	< 0.001	1.40	1.23-1.58	< 0.001
Complete high school	1.30	1.17-1.46	< 0.001	1.19	1.06-1.33	0.003
Incomplete higher education or more	1.00	-	-	1.00		
Level of monetary poverty			-			
Not poor	1.84	1.57-2.15	< 0.001	1.19	1.01-1.41	0.042
Not extreme poverty	1.49	1.26-1.77	< 0.001	1.13	0.95-1.35	0.173
Extreme poverty	1.00	-	-	1.00		
Food Insecurity Scale						
No food insecurity	2.23	1.83-2.71	< 0.001	2.00	1.61-2.47	< 0.001
Mild food insecurity	2.04	1.67-2.47	< 0.001	1.85	1.50-2.28	< 0.001
Moderate food insecurity	1.30	1.07-1.59	0.009	1.27	1.03-1.57	0.027
Severe food insecurity	1.00			1.00		
Parental emigration						
No	1.20	1.08-1.34	0.001	1.32	1.18-1.48	< 0.001
Yes	1.00	-		1.00		

95%CI: 95% confidence interval.

Source: *National Survey of Living Conditions* (ENCOVI 2021) ^9^.

## Discussion

Our study found that, among Venezuelan adults, worse self-rated health is associated with social and economic factors commonly described in the literature ^4^ ^,^ ^5^ ^,^ ^6^ ^,^ ^7^, such as being older, being a female individual, living in poverty, having a low level of education, and being in a food insecurity situation - with the first and last factors more strongly associated. Also important was the association of non-positive self-rated health with the emigration of a family member in the last five years, even when the factors were assessed independently.

Venezuela has a low percentage of adults who positively rate their own health (17.8%) when compared to estimates from neighboring countries (Argentina, Brazil, Chile, Colombia, Ecuador, Mexico, Peru, and Uruguay) ^21^. According to data collected between 2010 and 2014, in these countries, this indicator varies between 20% and 30%, except for Peru, which showed a prevalence of 46%. Relatively positive self-rated health in Venezuela has already been documented in prior studies. A population-based cohort study conducted between 2003 and 2007, which assessed 16,940 people aged 65 years and over in China, India, Cuba, the Dominican Republic, Peru, Mexico, Puerto Rico, and Venezuela, showed a higher prevalence of positive self-rated health in Venezuela, behind only rural China and urban India ^10^. In European countries, variation was observed in non-positive prevalence rates for self-assessed health, which were similar to those in Latin American countries, as reported in a study that assessed 11 European Union countries and Turkey ^7^, ranging from 49.2% in Portugal to 19.7% in Sweden. Direct comparisons must be carefully considered, as these countries have a higher proportion of older people when compared to Venezuela.

Given that the socioeconomic situation is a recognized determinant of the self-rated health ^4^, it was expected that a country in a serious economic crisis and with high levels of poverty and food insecurity would have a population with negative assessment of health status. In this sense, the results mentioned above raise doubts about the current theoretical paradigm of self-rated health and indicate that other factors, such as culture, may have an influence that is underreported in the literature.

Regarding the period during which ENCOVI 2021 was conducted (during the COVID-19 pandemic), studies have reported the impact of the health crisis on the self-rated health of individuals. In Brazil, a study found that a sedentary lifestyle and social distancing measures adopted during the pandemic contributed to a decline in self-rated health ^24^. In Venezuela, a study assessing 150 participants in the state of Mérida found that around 35% showed symptoms of stress, anxiety or depression during the pandemic ^25^. Of note, the ENCOVI 2021 did not include any specific question to assess the impact of the pandemic, which did not allow an assessment of self-rated health in the period.

A more negative self-rated health in women when compared to men is reported in studies from different countries ^5^ ^,^ ^7^ ^,^ ^26^ ^,^ ^27^ ^,^ ^28^, in agreement with the findings of our study. As observed in the adjusted analysis, Venezuelan women have a 35% higher prevalence of fair/poor self-rated health than Venezuelan men. Higher differences were reported in studies conducted in South Korea ^27^ (66%), Turkey ^7^ (55%), and India ^28^ (70%), although the last two used different self-rated health ratings, excluding the “fair” response. A Brazilian study that categorized self-rated health in a similar way to other studies showed that negative self-rated health among female individuals is smaller in the country, but still significant (23% higher among female individuals) ^5^.

The explanation for gender inequality in self-rated health varies in the literature. Some papers suggest the socially created association of being a man/being strong and being a woman/being fragile suggest men are healthier than women ^29^. Others highlight the role and position of women in society, which subjects them to double burden and disadvantages in their jobs ^30^. A higher prevalence of disabling chronic diseases of low lethality among women, such as arthritis and depression, is also highlighted as a possible explanation ^29^ ^,^ ^31^.

The results of our study confirm some findings from previous studies by highlighting the influence of age on self-perceived health. Studies suggest that aging is associated with a greater likelihood of negative self-rated health ^5^ ^,^ ^7^ ^,^ ^30^. Data from the *World Health Survey* in Brazil showed that increasing age reduces the chance of positive self-rated health in men and women, even after adjustment for socioeconomic variables ^30^. This trend can be explained by several factors. In general, younger people have fewer chronic health problems ^32^, which may contribute to a better self-rated health. However, Paskulin & Vianna ^33^ report that health perception is more related to the functional capabilities of individuals than the presence of chronic diseases.

As indicated in our study, there is an inverse relationship between educational level and non-positive health perception. Individuals with higher educational levels often describe their health positively ^4^ ^,^ ^5^ ^,^ ^7^ ^,^ ^34^. A study based on the *Health, Well-Being, and Aging in Latin America and the Caribbean* (SABE) survey ^34^ reported a complex relationship between education and self-rated health. Although self-rated health tends to improve as the educational level increases, this relationship is not linear. It is influenced by social opportunities, such as information, health services, and better living conditions. Alvarez-Galvez et al. ^4^, on the other hand, suggest that education has a stronger influence on self-rated health when compared to other socioeconomic indicators, such as income.

Monetary poverty, although related to non-positive self-rated health in the bivariate model, showed a weak association when other socioeconomic factors were taken into account. Income identification without a proper treatment, that is, used in absolute terms, is a fragile socioeconomic indicator ^35^. The idea is not what a person has, but what they can do with what they have. When incorporated into the concept of capabilities by Amartya Sen ^36^, it helps interpret the findings of our study. Although the identification of poverty in ENCOVI 2021 was based on estimated needs of Venezuelans ^22^, it is still based on income and not sufficient to identify the need of families. It explains, to a large extent, why the food insecurity situation has a stronger association with health status.

Food insecurity, unlike traditional socioeconomic indicators (income, education, and occupation), can identify disparities in demands and challenges between families ^6^ ^,^ ^37^. Marshall & Tucker-Seeley ^6^, when investigating the association of indicators related to specific forms of problems (struggle to pay bills, continuous financial stress, struggle to obtain medications, and food insecurity) with self-rated health, observed that all indicators were deeply associated, with food insecurity showing the strongest association. Most literature papers on food insecurity are focused on children, given their health is more severely impacted by lack of food ^38^. However, food insecurity is also associated with several health problems of the adult population, such as malnutrition, depression and other mental health problems, diabetes, hypertension, hyperlipidemia, and oral health problems ^38^.

Although the country has effective government policies on food security, the current economic and political crisis has had a significant negative impact on the access to proper food in the country ^39^. As observed in our study, the country has high levels of food insecurity, reaching 90% of the population. According to the report *The State of Food Security and Nutrition in the World* ^40^, the prevalence of malnutrition in the country increased significantly, from 8% between 2004 and 2006 to 23% between 2019 and 2021 , while the average in South American countries was 7% between 2019 and 2021. It indicates an alarming deterioration in food security in Venezuela in recent decades.

Emigration of a co-resident family member in the last five years was associated with non-positive self-rated health, also when other socioeconomic factors were taken into account. A recent study ^8^ that analyzed the health status of Venezuelan families that had emigration of a member reported that, in many cases, family members left in the country become responsible for the education and financial support of dependent minors, whether their own or of third parties, and for the care of dependent older people, a situation that often generates family and personal problems. According to the study, the prevalence of depression among these individuals was accentuated. An increase in unhealthy behaviors, such as alcohol and tobacco consumption, was also reported ^8^.

The serious economic crisis in Venezuela has affected the health infrastructure and the living and health conditions of its population ^14^. The Venezuelan health system has lost its operational capacity due to factors like lack of medications, vaccines, and basic health products; loss of emigrated health professionals; and unavailability of information (in particular since 2017) ^13^. From the population perspective of health conditions, several health indicators have significantly increased such as maternal and child mortality and the incidence of preventable diseases like HIV, tuberculosis, malaria, measles and diphtheria ^14^. In this context, our study provides reliable information about individuals whose health has had a more severe impact in the recent period.

On the other hand, some limitations should be considered. ENCOVI 2021 could not collect data during the COVID-19 pandemic, which led to many absent responses in some sections. However, the survey applied adjustments to minimize this problem, as explained in the technical report ^9^. Also, it is a cross-sectional study and cannot infer causality in the associations found, which requires careful interpretation of its findings.
