# Supplementary material for: Self-rated health and sociodemographic inequalities among Venezuelan adults: a study based on the National Survey of Living Conditions (ENCOVI 2021)
Source: Cad Saude Publica. 2024 Jun 21;40(6):e00149323. doi: 10.1590/0102-311XEN149323 (PMC11192572; doi:10.1590/0102-311XEN149323)
Supplement: Translation

# Translation: Autoavaliação de saúde e desigualdades sociodemográficas entre adultos venezuelanos: um estudo com base na *Pesquisa Nacional de Condições de Vida* (ENCOVI 2021)

**DOI:** 10.1590/0102-311XPT149323

**Authors:** Dalia Elena Romero, Anitza Freitez, Leo Ramos Maia, Nathalia Andrade de Souza

**Affiliations:** 1 Instituto de Comunicação e Informação Científica e Tecnológica em Saúde, Fundação Oswaldo Cruz, Rio de Janeiro, Brasil.; 2 Instituto de Investigaciones Económicas y Sociales, Universidad Católica Andrés Bello, Caracas, Venezuela.; 3 Escola Nacional de Saúde Pública Sérgio Arouca, Fundação Oswaldo Cruz, Rio de Janeiro, Brasil.

**Keywords:** Autoavaliação, Condição de Saúde, Inquéritos de Saúde

## Abstract

A autoavaliação de saúde é um indicador de simples captação em inquéritos de saúde, amplamente utilizado em pesquisas para medir aspectos físicos, sociais, mentais e de saúde da população, além de predizer a mortalidade precoce. No caso venezuelano, apenas recentemente começou a se coletar essa informação por meio da *Pesquisa Nacional de Condições de Vida* (ENCOVI). Nesse contexto, o estudo tem por objetivo analisar os fatores demográficos e socioeconômicos associados à autoavaliação não positiva da saúde entre adultos venezuelanos. Utiliza-se como fonte de dados a ENCOVI 2021 (n = 16.803), cuja amostra é probabilística e estratificada, apresentando perguntas sobre saúde, educação, migração e outros aspectos sociais e econômicos. Foram realizadas análises brutas e ajustadas de razão de prevalência, estimadas por meio de modelos de regressão de Poisson com variância robusta. A prevalência de autoavaliação da saúde regular/ruim entre venezuelanos foi de 17,8%. Os resultados indicaram uma forte associação entre a prevalência do desfecho e a faixa etária, sendo 3,81 vezes maior (IC95%: 3,29-4,41) entre os indivíduos com 60 anos ou mais, em comparação àqueles com idade de 18 a 29 anos. Além disso, os participantes em situação de insegurança alimentar severa apresentaram uma prevalência 2 vezes maior (IC95%: 1,61-2,47) do que aqueles que não enfrentaram nenhum nível de insegurança alimentar. Fatores como pobreza, escolaridade, emigração recente de familiares e sexo também demonstraram influência significativa, mesmo quando analisados independentemente. Os resultados destacam a necessidade de atenção especial à saúde daqueles que enfrentam fome e dos idosos.

## Introdução

A autoavaliação de saúde é um indicador de simples captação em inquéritos que expressa uma avaliação subjetiva do estado de saúde, mas que também serve de *proxy* das características objetivas da saúde dos indivíduos ^1^. Engloba tanto dimensões físicas quanto sociais, mentais e de saúde. Além disso, o indicador é utilizado como preditor da mortalidade prematura, o que despertou um considerável interesse na comunidade científica desde o final do século XX ^2^ ^,^ ^3^.

As circunstâncias sociais e econômicas têm impacto na nossa saúde física e mental, motivo pelo qual a relação da autoavaliação de saúde com fatores demográficos e socioeconômicos tem sido documentada ^4^ ^,^ ^5^ ^,^ ^6^ ^,^ ^7^. Embora essa relação varie em força entre os países ^4^, no geral, é bem estabelecida na literatura a associação de uma pior autoavaliação da saúde entre pessoas do gênero feminino ^5^ ^,^ ^7^, de idades avançadas ^5^ ^,^ ^7^, mais pobres ^4^ ^,^ ^6^ ^,^ ^7^, com baixa escolaridade ^4^ ^,^ ^5^ ^,^ ^7^ e em situação de insegurança alimentar ^6^.

Por outra parte, a influência da emigração familiar na autoavaliação de saúde em situações de crises migratórias tem sido subestimada. A maioria dos estudos sobre autoavaliação de saúde e migração foca na avaliação da condição dos emigrantes, negligenciando o impacto da migração nos membros da família que permanecem no país de origem ^8^. Estudos realizados nesses contextos de crise devem considerar que a migração fragmenta o núcleo familiar e gera uma maior responsabilidade dos cuidados familiares para um grupo menor de pessoas, o que pode afetar a saúde dos que permanecem.

No caso venezuelano, a coleta de informação sobre a autoavaliação de saúde começou apenas recentemente, com o inquérito nacional chamado *Encuesta Nacional de Condiciones de Vida* (ENCOVI; Pesquisa Nacional de Condições de Vida) ^9^, realizado durante a pandemia. Um estudo internacional sobre autoavaliação de saúde e que incluiu a Venezuela foi conduzido entre 2003 e 2007, mas se concentrou apenas na população idosa (n = 2.026) ^10^. A partir dele, os autores demonstraram que as pessoas idosas na Venezuela apresentavam uma autoavaliação de saúde favorável em comparação com oito outros países analisados ^7^.

Na Venezuela, a pobreza extrema tem se acentuado, passando de 9,3% a 76,6% entre 2013 e 2021 ^11^. A migração também tem sido intensa, entre 2,3 milhões e 4 milhões de emigrados venezuelanos desde 2015 ^12^. Nesse contexto de crise humanitária aguda, que também tem influenciado na deterioração do sistema de saúde ^13^, evidências sobre surtos de doenças infecciosas evitáveis ^14^, falta de vacinas e medicamentos ^13^ e internações e mortes por desnutrição ^15^ têm sido documentados. Tais achados indicam que as condições de saúde da população venezuelana têm piorado.

Há anos a Venezuela restringe a produção e divulgação da informação, inclusive epidemiológica ^12^. Para enfrentar o cerco das fontes de informação oficial, desde 2014 instituições de pesquisa venezuelanas passaram a realizar a ENCOVI ^9^. O estudo, de delineamento transversal, multitemático, cobertura nacional e periodicidade anual, tornou-se uma das fontes de informação mais utilizadas em artigos voltados às condições de vida e saúde da população venezuelana ^16^.

Conhecer a percepção subjetiva da saúde da população é fundamental em um país com sérios problemas econômicos, migratórios e de indisponibilidade de informação sobre saúde pública. Informação disponível, de qualidade, com regularidade e que dê respostas aos principais problemas da população é condição necessária para a obtenção de evidências científicas, para implantar ações efetivas na área da saúde e para monitorar as políticas públicas ^17^. Nesse sentido, este artigo tem por objetivo analisar os fatores demográficos e socioeconômicos associados à autoavaliação da saúde não positiva entre adultos venezuelanos.

## Métodos

### Fontes de dados e amostra

A Venezuela é um país localizado na região norte da América do Sul. As projeções do Escritório de População da Organização das Nações Unidas (ONU) apontam que, em 2021, a Venezuela contabilizava 28.436.000 habitantes. O país enfrentou desafios econômicos e políticos significativos nos últimos anos, o que impactou sua situação socioeconômica e demográfica ^16^.

Este estudo tem desenho transversal de base populacional, realizado com dados da pesquisa ENCOVI, conduzido na Venezuela entre os meses de fevereiro e abril de 2021, e coordenado pelo Instituto de Investigações Econômicas e Sociais (IIES) da Universidade Católica Andrés Bello (UCAB). A ENCOVI 2021 contempla perguntas relativas à educação, saúde, nutrição e acesso à alimentos, pobreza, desemprego e migração da população residente na Venezuela.

A amostragem do inquérito foi probabilística por conglomerado e em três estágios, estimada com base na renda familiar *per capita*, obtida por meio da ENCOVI 2019-2020, a nível estadual. Seu questionário contava com 21 seções e 36 subseções e, no total, tinha 788 perguntas. As perguntas foram feitas por lares e para todos os membros do domicílio. Mais informações sobre a ENCOVI 2021 podem ser acessadas por meio do documento técnico da pesquisa ^9^.

A amostra final, composta por 42.444 pessoas, foi calibrada com pesos que consideraram os dados do censo de 2011 e as projeções populacionais de 2021, realizadas pela revisão do *World Population Prospects* (Perspectivas da População Mundial), da ONU.

Este estudo considerou os registros de indivíduos adultos com idade ≥ 18 anos e que responderam à pergunta: “Nos últimos 12 meses, como você acha que está o estado de saúde do entrevistado?” (n = 16.803).

### Variáveis

O desfecho de interesse neste estudo foi a autoavaliação não positiva da saúde. Para sua obtenção, foi utilizada a pergunta “Nos últimos 12 meses, como você acha que está o estado de saúde do entrevistado?”, que tinha como categorias de resposta: “muito bom”, “bom”, “regular” ou “ruim”. As duas últimas categorias foram agrupadas para a definição do desfecho. Essa categorização é comumente utilizada em estudos semelhantes, o que possibilita a comparação entre eles ^6^ ^,^ ^18^ ^,^ ^19^ ^,^ ^20^ ^,^ ^21^.

As características demográficas associadas ao desfecho foram o sexo (homem ou mulher) e as faixas etárias relativas aos adultos jovens (18 a 29 anos), adultos (30 a 59 anos) e idosos (60 anos e mais).

Os fatores socioeconômicos considerados neste estudo foram:

Escolaridade, cuja pergunta “Qual foi o último nível educacional em que o entrevistado obteve aprovação de série, ano, semestre ou trimestre? foi categorizada em: ensino primário completo ou menos; ensino médio incompleto; ensino médio completo; e ensino superior incompleto ou completo;

Nível de pobreza monetária estimada pela ENCOVI 2021 a partir da renda *per capita* domiciliar relacionada ao valor normativo da cesta básica normativa (Canasta Alimentaria Normativa - CAN), cujo valor cobre as necessidades calóricas mínimas ^22^. Nos casos em que a renda *per capita* é maior ou igual a duas vezes o valor da CAN *per capita*, o entrevistado é considerado “não pobre”. Se a renda *per capita* for maior ou igual ao valor do CAN, mas menor que o dobro do seu valor, considera-se “pobre não extremo”. Se a renda *per capita* do entrevistado é menor que o valor da CAN, considera-se “extremamente pobre”;

Escala de Insegurança Alimentar estimada na ENCOVI 2021 com base na *Escala Latino-americana e Caribenha de Segurança Alimentar* (Escala Latinoamericana y Caribeña de Seguridad Alimentaria - ELCSA) ^23^. A ELCSA é constituída a partir de 15 questões que se referem a situações que os entrevistados enfrentaram durante os três meses anteriores à data da entrevista, relacionadas com a quantidade e a qualidade dos alimentos disponíveis e com as estratégias que utilizam em busca de aliviar as carências alimentares. As categorias contemplam: insegurança alimentar severa; insegurança alimentar moderada; insegurança alimentar leve e sem insegurança alimentar. Aqueles com insegurança alimentar severa foram considerados em situação de fome;

Migração parental definida como a emigração recente de um familiar corresidente, identificada a partir da pergunta “Durante os últimos 5 anos, desde janeiro de 2016, alguma pessoa que vive ou viveu com você em sua residência foi morar em outro país?”, a qual tinha como possibilidades de respostas sim ou não.

### Análise

Inicialmente, realizou-se a caracterização da população estudada segundo as variáveis consideradas no estudo por meio da distribuição percentual e respectivos intervalos de 95% de confiança (IC95%). Em seguida, estimaram-se as prevalências de autoavaliação de saúde regular/ruim segundo características demográficas e socioeconômicas e a associação entre as variáveis foi verificada pelo teste qui-quadrado de Pearson com correção de Rao-Scott, considerando-se um nível de significância de 5%.

Na análise dos fatores independentemente associados à autoavaliação não positiva da saúde, foram consideradas as variáveis que apresentaram associação significativa com o desfecho na análise bivariada, sendo elas: sexo, faixa etária, escolaridade, nível de pobreza monetária, insegurança alimentar e migração parental. Foram estimadas as razões de prevalências (RP) e IC95% por meio de modelos de regressão de Poisson com variância robusta. As análises foram realizadas no pacote estatístico SPSS 21 (https://www.ibm.com/), considerando o peso amostral obtido para calibração da amostra.

### Aspectos éticos

Por ser uma pesquisa que utiliza exclusivamente dados secundários de domínio público está dispensada de submissão e aprovação por Comitê de Ética de Pesquisa com Seres Humanos, de acordo com a *Resolução nº 466/2012*, do Conselho Nacional de Saúde.

## Resultados

A população do estudo foi predominantemente feminina (64,8%; IC95%: 64,0-65,5) e de pessoas com 30 a 59 anos de idade (60,3%; IC95%: 59,5-61,1) (Tabela 1).

**Tabela 1:** Distribuição percentual da população adulta venezuelana segundo características demográficas e socioeconômicas. Venezuela, 2021.

Variáveis	n	%	IC95%
Sexo			
Masculino	5.471	35,2	34,5-36,0
Femininor	10.066	64,8	64,0-65,5
Faixa etária (anos)			
18-29	2.895	18,6	18,0-19,2
30-59	9.368	60,3	59,5-61,1
60 ou mais	3.275	21,1	20,4-21,7
Escolaridade			
Sem escolaridade	60	0,4	0,3-0,5
Primário incompleto	1.505	10,1	9,6-10,6
Primário completo	2.187	14,7	14,1-15,3
Médio incompleto	2.321	15,6	14,7-16,2
Médio completo	4.642	31,2	30,5-32
Superior técnico (incompleto ou completo)	1.184	8,0	7,5-8,4
Superior universitário (incompleto ou completo)	2.963	19,9	19,3-20,6
Nível de pobreza monetária			
Não pobre	1.317	8,5	8,1-9,5
Pobreza não extrema	3.480	22,6	21,9-23,2
Pobreza extrema	10.631	68,9	68,2-69,6
Escala de Insegurança Alimentar			
Sem insegurança alimentar	1.093	7,0	6,6-7,4
Insegurança alimentar leve	5.264	33,9	33,2-35,1
Insegurança alimentar moderada	5.172	33,3	32,6-34,1
Insegurança alimentar severa	3.992	25,7	25,0-26,4
Migração parental			
Não	14.105	90,8	90,4-91,3
Sim	1.425	9,2	8,7-9,6
Autoavaliação da saúde			
Muito boa	6.011	38,7	38,0-39,4
Boa	6.763	43,5	42,9-44,2
Regular	2.554	16,4	16,0-16,9
Ruim	209	1,3	1,2-1,5

IC95%: intervalo de 95% de confiança.

Fonte: *Pesquisa Nacional de Condições de Vida* (ENCOVI-2021) ^9^.

A precária condição socioeconômica de grande parte da população venezuelana é evidenciada pelos resultados da Tabela 1. Mais de 90% estavam em algum nível de pobreza monetária ou de insegurança alimentar. Destacam-se os casos extremos: quase 7 em cada 10 venezuelanos se encontravam em extrema pobreza (68,9%; IC95%: 68,2-69,6), enquanto 25,7% (IC95%: 25,0-26,4) enfrentavam a insegurança alimentar severa, ou seja, estavam passando fome.

Cerca de 80% dos adultos venezuelanos avaliaram sua saúde positivamente, com 38,7% (IC95%: 38,0-39,4) classificando-a como muito boa e 43,5% (IC95%: 42,9-44,2) como boa (Tabela 1).

A prevalência da autoavaliação de saúde regular/ruim de acordo com as características demográficas e socioeconômicas analisadas são apresentadas na Tabela 2. Do total de venezuelanos adultos, 17,8% (IC95%: 17,2-18,4) relataram autoavaliação de saúde regular/ruim. Os grupos que mais frequentemente avaliaram sua saúde negativamente foram: pessoas idosas (32,9%; IC95%: 31,3-34,5); pessoas que, no máximo, completaram o ensino primário (24,6%; IC95%: 23,2-25,9); pessoas com insegurança alimentar severa (23,8%; IC95%: 22,5-25,1); e pessoas com familiares emigrados recentemente (22,7%; IC95%: 20,5-24,9). Por outro lado, jovens adultos e pessoas sem insegurança alimentar apresentaram prevalência de autoavaliação de saúde regular/ruim abaixo de 10% (Tabela 2).

**Tabela 2:** Prevalência de venezuelanos adultos com autoavaliação da saúde regular/ruim, segundo características demográficas e socioeconômicas. Venezuela, 2021.

Variáveis	%	IC95%	Valor de p
Total	17,8	17,2-18,4	
Sexo			< 0,001
Masculino	14,9	14,0-15,8	
Feminino	19,4	18,6-20,1	
Faixa etária (anos)			< 0,001
18-29	7,7	6,7-8,7	
30-59	15,6	14,9-16,3	
60 ou mais	32,9	31,3-34,5	
Escolaridade			< 0,001
Primário completo ou menos	24,6	23,2-25,9	
Médio incompleto	19,1	17,5-20,7	
Médio completo	15,0	13,9-16,0	
Superior incompleto ou mais	12,3	11,3-13,3	
Nível de pobreza monetária			< 0,001
Não pobre	12,6	10,8-14,4	
Pobreza não extrema	17,0	15,8-18,2	
Pobreza extrema	18,7	17,9-19,4	
Escala de Insegurança Alimentar			< 0,001
Sem insegurança alimentar	9,4	7,7-11,2	
Insegurança alimentar leve	12,7	11,8-13,6	
Insegurança alimentar moderada	20,0	18,9-21,1	
Insegurança alimentar severa	23,8	22,5-25,1	
Migração parental			< 0,001
Não	17,3	16,7-17,9	
Sim	22,7	20,5-24,9	

IC95%: intervalo de 95% de confiança.

Fonte: *Pesquisa Nacional de Condições de Vida* (ENCOVI-2021) ^9^.

Na análise dos fatores independentemente associados à autoavaliação não positiva da saúde (Tabela 3), observou-se que ser mulher, ter mais idade, menor escolaridade, maior nível de pobreza e insegurança alimentar e ter um familiar que emigrou em período recente foram fatores associados a esse desfecho. Na análise ajustada, dois fatores se destacaram como os mais associados à autoavaliação de saúde regular/ruim: idade e insegurança alimentar; entre os idosos, a percepção negativa da saúde foi cerca de quatro vezes maior em relação aos mais jovens (RP = 3,81; IC95%: 3,29-4,41) e, naqueles indivíduos em situação de insegurança alimentar severa, essa percepção foi duas vezes maior em comparação aos que não enfrentavam nenhum nível de insegurança alimentar (RP = 2,00; IC95%: 1,61-2,47).

**Tabela 3:** Razões de prevalência (RP) brutas e ajustadas da autoavaliação da saúde regular/ruim, segundo características demográficas e socioeconômicas em pessoas adultas. Venezuela, 2021.

Variáveis	RP bruta	IC95%	Valor de p	RP ajustada	IC95%	Valor de p
Sexo						
Masculino	1,41	1,31-1,53	< 0,001	1,35	1,25-1,47	< 0,001
Feminino	1,00	-	-	1,00		
Faixa etária (anos)						
18-29	4,10	3,57-4,71	< 0,001	3,81	3,29-4,41	< 0,001
30-59	1,97	1,72-2,25	< 0,001	1,91	1,66-2,20	< 0,001
60 ou mais	1,00	-	-	1,00		
Escolaridade						
Primário completo ou menos	1,45	1,31-1,62	< 0,001	1,36	1,22-1,53	< 0,001
Médio incompleto	1,57	1,39-1,76	< 0,001	1,40	1,23-1,58	< 0,001
Médio completo	1,30	1,17-1,46	< 0,001	1,19	1,06-1,33	0,003
Superior incompleto ou mais	1,00	-	-	1,00		
Nível de pobreza monetária			-			
Não pobre	1,84	1,57-2,15	< 0,001	1,19	1,01-1,41	0,042
Pobreza não extrema	1,49	1,26-1,77	< 0,001	1,13	0,95-1,35	0,173
Pobreza extrema	1,00	-	-	1,00		
Escala de Insegurança Alimentar						
Sem insegurança alimentar	2,23	1,83-2,71	< 0,001	2,00	1,61-2,47	< 0,001
Insegurança alimentar leve	2,04	1,67-2,47	< 0,001	1,85	1,50-2,28	< 0,001
Insegurança alimentar moderada	1,30	1,07-1,59	0,009	1,27	1,03-1,57	0,027
Insegurança alimentar severa	1,00			1,00		
Migração parental						
Não	1,20	1,08-1,34	0,001	1,32	1,18-1,48	< 0,001
Sim	1,00	-		1,00		

IC95%: intervalo de 95% de confiança.

Fonte: *Pesquisa Nacional de Condições de Vida* (ENCOVI-2021) ^9^.

## Discussão

Este estudo verificou que, na população adulta venezuelana, a pior autoavaliação de saúde está associada a fatores sociais e econômicos comumente descritos na literatura ^4^ ^,^ ^5^ ^,^ ^6^ ^,^ ^7^, tais como ser idoso, mulher, estar em situação de pobreza, ter baixo nível de escolaridade e estar em situação de insegurança alimentar, sendo o primeiro e o último fatores aqueles com maior força de associação. Destaca-se, ainda, a associação da autoavaliação não positiva da saúde com a emigração de um familiar nos últimos cinco anos, mesmo quando considerados os fatores de forma independente.

A Venezuela apresenta um baixo percentual de adultos que avaliam a própria saúde de forma não positiva (17,8%), se comparado a estimativas de países vizinhos (Argentina, Brasil, Chile, Colômbia, Equador, México, Peru e Uruguai) ^21^. Segundo dados de inquéritos coletados entre 2010 e 2014, esse indicador varia, nesses países, entre 20 e 30%, à exceção do Peru, que apresentou prevalência de 46%. A situação relativamente positiva da autoavaliação de saúde na Venezuela foi documentada em pesquisas anteriores. Estudo de coorte de base populacional realizado entre 2003 e 2007 ^10^, que incluiu 16.940 pessoas com idade de 65 anos ou mais na China, Índia, Cuba, República Dominicana, Peru, México, Porto Rico e Venezuela, mostrou que este último tinha alta prevalência de autoavaliação de saúde positiva, atrás apenas da China rural e da Índia urbana. Nos países europeus, observaram-se variações nas prevalências não positivas para a saúde autoavaliada semelhantes à dos países latino-americanos, conforme verificado em estudo que a estimou para 11 países da União Europeia e para a Turquia ^7^, variando de 49,2% em Portugal a 19,7% na Suécia. Ressalta-se que a comparação direta deve ser cuidadosa, considerando que esses países apresentam maior proporção de pessoas idosas do que a Venezuela.

Tendo em vista que a situação socioeconômica é um reconhecido determinante para a autoavaliação de saúde ^4^, esperava-se que um país em profunda crise econômica, com altos níveis de pobreza e insegurança alimentar, tivesse uma população que avaliasse negativamente seu estado de saúde. Nesse sentido, os resultados supracitados levantam dúvidas sobre o atual paradigma teórico da autoavaliação de saúde e indicam que outros fatores, como a cultura, talvez tenham influência subestimada na literatura.

No que se refere ao período de realização da ENCOVI 2021 - durante a pandemia de COVID-19 -, estudos têm mostrado um impacto da crise sanitária na autoavaliação de saúde dos indivíduos. No Brasil, estudo apontou que o sedentarismo e as medidas de distanciamento social adotadas durante a pandemia foram fatores que contribuíram para uma piora na autoavaliação de saúde ^24^. Já na Venezuela, uma pesquisa realizada com 150 participantes no Estado de Mérida constatou que aproximadamente 35% deles apresentaram sintomas de estresse, ansiedade ou depressão durante a pandemia ^25^. Destaca-se que a pesquisa ENCOVI 2021 não usou perguntas específicas para avaliar o impacto da pandemia, impossibilitando uma avaliação relacionada à autoavaliação de saúde no período.

A pior autoavaliação de saúde de mulheres, em comparação aos homens, é verificada em estudos de diversos países ^5^ ^,^ ^7^ ^,^ ^26^ ^,^ ^27^ ^,^ ^28^, evidências que vão ao encontro dos achados deste estudo. Conforme demonstrado na análise ajustada, venezuelanas apresentam prevalência de autoavaliação de saúde regular/ruim 35% maior do que venezuelanos. Diferenciais maiores foram encontrados em estudos voltados à Coreia do Sul ^27^ (66%), à Turquia ^7^ (55%) e à Índia ^28^ (70%), embora os dois últimos tenham categorizado a autoavaliação de saúde de forma diferente, excluindo a categoria regular do desfecho. Estudo brasileiro que categorizou a autoavaliação de saúde de forma semelhante a esses demonstrou que a desvantagem feminina no país é menor, porém significativa (23% maior para mulheres) ^5^.

A explicação para a desigualdade de gênero na autoavaliação de saúde varia na literatura. Alguns sugerem que a associação, criada socialmente, do ser homem/ser forte e ser mulher/ser frágil, os sujeitam a se apresentarem como mais saudáveis do que as mulheres ^29^. Outros destacam o papel e a posição da mulher na sociedade, que a condiciona a uma dupla jornada de trabalho e a desvantagens no mercado de trabalho remunerado ^30^. A maior prevalência de doenças crônicas incapacitantes e com baixa letalidade entre mulheres, como a artrite e a depressão, também é apontada como possível explicação ^29^ ^,^ ^31^.

Os resultados deste estudo corroboram com achados de pesquisas anteriores ao evidenciar a influência da idade na autopercepção de saúde. Estudos apontam que o envelhecimento está associado a uma maior probabilidade de autoavaliação de saúde negativa ^5^ ^,^ ^7^ ^,^ ^30^. Dados da *Pesquisa Mundial da Saúde*, no Brasil, destacaram que o aumento da idade diminui a chance de autoavaliação de saúde positiva em homens e mulheres, mesmo após o ajuste por variáveis socioeconômicas ^30^. Essa tendência pode ser explicada por diversos fatores. Pessoas mais jovens geralmente têm menos problemas de saúde crônicos ^32^, o que pode contribuir para uma melhor autoavaliação de saúde. Não obstante, Paskulin & Vianna ^33^ pontuam que a percepção de saúde está mais relacionada às capacidades funcionais dos indivíduos do que à presença de doenças crônicas.

Como indicado neste estudo, há uma relação inversa entre o nível de escolaridade e a percepção não positiva da saúde. Indivíduos com níveis educacionais mais elevados frequentemente descrevem sua saúde de forma positiva ^4^ ^,^ ^5^ ^,^ ^7^ ^,^ ^34^. Estudo baseado nos dados da pesquisa *Saúde, Bem-estar e Envelhecimento na América Latina e Caribe* (SABE) ^34^ revela uma relação complexa entre a escolaridade e a autoavaliação de saúde. Embora haja uma tendência de melhora na autoavaliação de saúde à medida que a escolaridade aumenta, essa relação não é linear. Ela é influenciada pelas oportunidades sociais, como informação, serviços de saúde e melhores condições de vida. Alvarez-Galvez et al. ^4^, por outra parte, sinalizam que a escolaridade determina em maior grau a autoavaliação de saúde do que outros indicadores socioeconômicos, como a renda.

A pobreza monetária, embora tenha se mostrado associada à autoavaliação não positiva da saúde no modelo bivariado, apresentou uma associação fraca quando considerados os demais fatores socioeconômicos. A captação e identificação da renda, sem um adequado tratamento, ou seja, utilizada em termos absolutos, é um indicador socioeconômico frágil ^35^. O fundamental não é o que uma pessoa tem, mas sim o que ela pode fazer com isso. Essa ideia, incorporada ao conceito de “*capabilities*” de Amartya Sen ^36^, é esclarecedora para a interpretação dos achados deste estudo. Embora a captação da pobreza na ENCOVI 2021 tenha sido feita com base em uma estimativa das necessidades do povo venezuelano ^22^, ela, ainda assim, é baseada em termos de renda, não sendo suficiente para identificar a situação de carência das famílias. Isso explica, em grande medida, o porquê da situação de insegurança alimentar apresentar associação mais forte com o estado de saúde.

A insegurança alimentar, diferentemente dos indicadores socioeconômicos tradicionais (renda, escolaridade e ocupação), é capaz de captar as disparidades nas demandas e dificuldades entre famílias ^6^ ^,^ ^37^. Marshall & Tucker-Seeley ^6^, com o objetivo de investigar a associação de indicadores relacionados a formas específicas de dificuldades (dificuldade para pagar contas, estresse financeiro contínuo, dificuldade na obtenção de medicamentos e insegurança alimentar) com a autoavaliação de saúde, observaram que todos os indicadores estavam fortemente associados, sendo a insegurança alimentar a que apresentou maior força de associação. A maior parte da literatura voltada à insegurança alimentar concentra-se nas crianças, haja vista que a saúde delas é impactada de forma mais severa pela escassez de alimentos ^38^. No entanto, a insegurança alimentar também está associada a diversos problemas de saúde na população adulta, tais como: desnutrição, diabetes, hipertensão, hiperlipidemia, problemas de saúde bucal, depressão e outros problemas de saúde mental ^38^.

Embora o país tenha sido reconhecido por suas políticas governamentais eficazes em segurança alimentar, a atual crise econômica e política tem impactado negativamente o acesso à alimentação adequada no país ^39^. Como evidenciado neste estudo, atualmente a insegurança alimentar tem altos níveis no país, atingindo 90% da população. De acordo com o relatório sobre o estado da segurança alimentar e nutricional no mundo (*The State of Food Security and Nutrition in the World*) ^40^, a prevalência de desnutrição no país aumentou significativamente - de 8%, entre 2004 e 2006, para 23%, entre 2019 e 2021 -, enquanto a média dos países da América do Sul foi de 7% nesse último período. Isso indica uma preocupante deterioração da segurança alimentar na Venezuela nas últimas décadas.

A emigração de um familiar corresidente nos últimos cinco anos esteve associada à autoavaliação não positiva da saúde, mesmo quando levado em consideração os demais fatores socioeconômicos. Estudo recente ^8^ analisou o estado de saúde das famílias venezuelanas que sofreram a emigração de alguns de seus membros e aponta que, em muitos casos, os familiares “abandonados” no país passam a se responsabilizar pela educação e sustento de menores dependentes, sejam próprios ou de terceiros, e pelos cuidados de pessoas idosas dependentes, situação que costuma gerar problemas familiares e pessoais. De acordo com o estudo, a prevalência de depressão entre esses indivíduos foi acentuada. O aumento de comportamentos prejudiciais à saúde, como o consumo de álcool e tabaco, também foi relatado ^8^.

A grave crise econômica da Venezuela tem afetado a infraestrutura sanitária e as condições de vida e de saúde de sua população ^14^. O sistema de saúde venezuelano vem perdendo a sua capacidade operacional devido a fatores como: a escassez de medicamentos, vacinas e produtos básicos de saúde; a perda de profissionais de saúde emigrados; e a indisponibilidade de informações (especialmente a partir de 2017) ^13^. Na perspectiva das condições de saúde da população, observa-se um aumento vertiginoso em diversos indicadores de saúde, entre os quais se destacam: a mortalidade materna e infantil; e a incidência de doenças evitáveis, como HIV, tuberculose, malária, sarampo e difteria ^14^. Nesse contexto, este estudo se destaca por fornecer informações confiáveis acerca daqueles que sofreram maior impacto em sua saúde no período recente.

Por outro lado, algumas limitações devem ser consideradas. A ENCOVI 2021 enfrentou dificuldades para coletar dados presencialmente na pandemia de COVID-19, o que gerou falta de respostas em algumas áreas. Porém, a pesquisa aplicou ajustes para minimizar esse problema, conforme explicado no relatório técnico ^9^. Além disso, o estudo é transversal e não pode inferir causalidade nas associações encontradas, o que requer uma interpretação cuidadosa dos achados.
